# Patients’ Use of Electronic Health Records Facilitates Patient-Centered Communication: Findings From the 2017 Health Information National Trends Survey

**DOI:** 10.2196/50476

**Published:** 2024-11-25

**Authors:** Suhwoo Ahn, Chul-joo Lee, Inhwan Bae

**Affiliations:** 1 Hubbard School of Journalism and Mass Communication University of Minnesota, Twin Cities Minneapolis, MN United States; 2 Department of Communication Seoul National University Gwanak-gu, Seoul Republic of Korea; 3 Department of Communication Cornell University Ithaca, NY United States

**Keywords:** electronic health record, health information efficacy, patient-centered communication, social support, patient-centered care

## Abstract

**Background:**

Patient-centered communication refers to interaction between patients and health professionals that considers patients’ preferences and empowers patients to contribute to their own care. Research suggests that patient-centered communication promotes patients’ satisfaction with care, trust in physicians, and competence in their abilities to manage their health.

**Objective:**

The study aims to explore the role of patients’ use of electronic health records (EHRs) in promoting patient-centered communication. Specifically, we investigated how health information efficacy mediates the association of EHR use with patient-centered communication and whether and how the relationship between EHR use and health information efficacy varies according to patients’ perceived social support levels.

**Methods:**

We conducted mediation and multigroup analyses using nationally representative data from the Health Information National Trends Survey 5 cycle 1 conducted in the United States (N=3285). Among respondents, we analyzed those who received care from health professionals over the previous year (2823/3285, 85.94%).

**Results:**

EHR use by patients was associated with high levels of health information efficacy (unstandardized coefficient=0.050, SE 0.024; *P*=.04). In turn, health information efficacy was positively related to patient-centered communication (unstandardized coefficient=0.154, SE 0.024; *P*<.001). The indirect pathway from EHR use to patient-centered communication, mediated by health information efficacy, was statistically significant (unstandardized coefficient=0.008, SE 0.004; *P*=.04). Among patients with high social support (2349/2823, 83.21%), EHR use was not significantly associated with health information efficacy (unstandardized coefficient=0.038, SE 0.026; *P*=.15), although health information efficacy was linked to high levels of patient-centered communication (unstandardized coefficient=0.151, SE 0.030; *P*<.001). The indirect relationship in this group was not significant (unstandardized coefficient=0.006, SE 0.004; *P*=.11). However, among those with low social support (474/2823, 16.79%), EHR use was positively associated with health information efficacy (unstandardized coefficient=0.155, SE 0.048; *P*=.001), which in turn relates to high levels of patient-centered communication (unstandardized coefficient=0.137, SE 0.050; *P*=.01). The indirect pathway was also significant (unstandardized coefficient=0.021, SE 0.010; *P*=.03).

**Conclusions:**

Patients who use EHRs may build health information efficacy, which seems to promote communication between patients and health care providers. This indirect pathway was not detected among patients with high social support. However, among those with low social support, EHR use seems to enhance health information efficacy, which may in turn facilitate patient-centered communication. Given the nature of the dataset used, the findings of this study are more relevant to the United States than other contexts.

## Introduction

### Background

Current health care aims to promote patient-centeredness as a key element of high-quality care [1]. A primary goal of patient-centered care is patient-centered communication [2]. Patient-centered communication refers to interaction between patients and health professionals that considers patients’ preferences and empowers patients to contribute to their own care [1,3]. Specifically, patient-centered communication is defined by its functions: fostering relationships between patients and health professionals, exchanging information, responding to patients’ emotional reactions, assisting patients in relieving uncertainty, involving patients with medical decision-making, and encouraging patients to manage their health [2-4]. Research suggests that patient-centered communication promotes patients’ satisfaction with care [5], trust in physicians [6,7], and competence in their abilities to manage their health [8]. In addition, patient-centered communication plays a crucial role in preventing patients from believing health misinformation [9,10]. Because health misinformation is rampant on social media platforms [10,11] and misinformation is likely to be disseminated more rapidly than corrective information [12], health professionals today use various strategies to counter patients’ misinformed beliefs [9].

Emerging evidence indicates that patients’ use of electronic health records (EHRs) has the potential to promote patient-centered communication [4,13-17]. An EHR is an electronic version of a patient’s personal health information that contains health conditions and medication history [4,18-20]. Health care providers generate health records, and patients can access this information from EHRs [4,18,19]. To our knowledge, however, no studies have investigated the underlying mechanism between EHR use and patient-centered communication.

This study suggests that patients’ confidence in their abilities to obtain health information they want, termed health information efficacy [21], may play a role here. Because EHR use empowers patients to control their health status [16], we argue that EHR use is positively linked to health information efficacy, which may in turn be related to patient-centered communication. Moreover, we posit that EHR use is beneficial to patients with little social support. Given that EHRs may compensate for patients’ lack of social support, we postulate that the relationship between use of EHRs and health information efficacy might be strong for patients who perceive little social support.

### The Development of EHRs

EHRs and electronic medical records (EMRs) are the 2 main computerized systems used to share patient information [19]. Although EHRs and EMRs are sometimes used interchangeably, they are distinct from each other [22]. EMRs were developed to deliver and receive patient information among clinicians within a particular hospital [20,22,23]. Unlike an EMR system, EHRs allow health professionals to share patient information with health care providers in other institutions [19,20,22]. Moreover, EHRs are designed to be used by both health professionals and patients [19,20,22,23]. Thus, patients who use EHRs are able to access their health records, obtain health information, and communicate with health professionals [14,15,19,24].

While EHRs and EMRs are typically controlled by physicians, personal health records (PHRs) are primarily managed by patients themselves, and various entities, such as physicians, patients, and pharmacies, could enter information on PHRs [20,25,26]. That is, PHRs serve as a compilation of individual patients’ health information over their lifetime [26]. Although PHRs allow patients to organize their own health information logically, health professionals raise concerns regarding the inaccuracy of health information within PHRs [20]. Therefore, some scholars argue that EHRs need to be combined with PHRs [26,27].

### The Relationship Between the Use of EHRs and Patient-Centered Communication

It is not always easy for patients to get useful health information from the media and interpersonal sources. Understanding medical terminologies or jargon that health care professionals use requires basic health literacy and cognitive abilities [28]. Although the traditional media and the internet deliver a wealth of information, patients should spend much time finding proper health information [29]. In addition, not all patients have friends or family members who could provide useful health information [30,31].

However, EHRs enable patients to easily access health information they need [15,17,19,24]. By granting patients access to their personal medical history, including treatment plans, medications, radiology images, allergy information, laboratory results, and immunization dates [32], patients who use EHRs can obtain health information tailored to their needs and circumstances [14,15]. In addition, patients can efficiently communicate with health care professionals through the messaging features within EHR platforms [14,33]. For example, patients are able to send quick messages to health professionals and receive immediate responses through EHRs whenever they have questions about their health status [14,18]. Consequently, patients who use EHRs may perceive that acquiring necessary health information is not as difficult.

Self-efficacy theory posits that people who experience success develop confidence in their capabilities to attain goals [34]. In a health context, patients who acquire useful health information build health information efficacy, which refers to one’s confidence in their abilities to get the health information they want [21]. This study proposes that EHR use may enhance health information efficacy. Patients who use EHRs are able to cultivate experiences of successfully obtaining the health information they need by accessing their personal health information, such as laboratory results or medications stored within EHRs [14]. Through this process, patients may come to realize that it is not as difficult to obtain personal health information from EHRs. Moreover, enhanced confidence may lead patients to believe that acquiring general health information from other information sources, such as the media or interpersonal networks, is also less challenging than previously thought. In other words, continued access to personal health information from EHRs may foster patients’ confidence in their abilities to obtain general health information from various information sources beyond EHRs.

Therefore, we advance the following hypothesis: EHR use will be positively related to health information efficacy (hypothesis 1).

Patients’ efficacy beliefs about acquiring health information may be applied to situations where patients interact with health professionals. That is, patients with health information efficacy will have the confidence that they can acquire information from health care providers as well. Such confidence may allow patients to communicate efficiently with health care professionals for the following reasons. First, patients with health information efficacy will seek information and prepare a list of well-informed questions before consulting with physicians [35]. Physicians are likely to offer more information to patients who ask many questions than to those with few questions [36,37]. In the process of asking questions and getting answers, patients will communicate actively with their physicians. Second, patients with efficacy beliefs about obtaining health information may learn about their health while interacting with their physicians. Patients who are well-informed are likely to engage in shared decision-making with health professionals [38]. Accordingly, it is expected that patients with health information efficacy have patient-centered communication with health care professionals.

Hence, we posit the following hypothesis: health information efficacy will be positively related to patient-centered communication (hypothesis 2).

Taken together, it is proposed that health information efficacy will mediate the linkage between EHR use and patient-centered communication (hypothesis 3).

### The Moderating Role of Social Support in Using EHRs

Social support is defined as individuals’ perceptions that they have someone to rely on [39-41]. People feel supported when receiving assistance from significant others, such as family members, friends, or coworkers [40,42]. In a health context, supportive significant others provide patients with health information, such as coping strategies for stress [40] or advice on how to care for infants [43]. Moreover, social support provides patients with a source of efficacy beliefs about obtaining and understanding health information [41].

Research has shown that eHealth, defined as the use of information and communication technologies to improve health care [44-46], has the potential to assist patients in overcoming deficits in social support [47-50]. In this study, we argue that EHRs, a type of eHealth service, may compensate for patients’ lack of social support.

EHRs may enable patients with little social support to build efficacy beliefs about obtaining health information. Patients who lack social support are less likely to have supportive others who offer essential health information and emotional support [40,48]. Those patients may receive useful health information through EHR use [15] because EHRs provide patients with health information in an edited form that is easier to understand [51]. In addition, patients can ask questions through EHRs whenever they encounter complicated terms in their medical records [14], which makes them feel comfortable about their care. Naturally, patients who lack social support will cultivate successful experiences of acquiring health information through EHRs. Such mastery experiences may develop patients’ efficacy beliefs about getting health information. Thus, among patients with low social support, the linkage between the use of EHRs and health information efficacy may be strong.

On the contrary, the relationship between EHR use and health information efficacy may be weak among individuals with high social support. Because social support increases patients’ confidence in obtaining and understanding health information [41], patients with high social support may already have a sufficient level of health information efficacy. Therefore, individuals with high levels of social support may gain limited benefits from EHR use when it comes to building confidence in their ability to acquire health information. That is, a ceiling effect may occur.

Thus, EHR use may narrow the gap in health information efficacy between those with high and low social support. Accordingly, we suggest the following hypothesis: the association of EHR use with health information efficacy will be stronger among those with low social support than those with high social support (hypothesis 4).

## Methods

### Study Participants

This study analyzed nationally representative survey data from cycle 1 of the fifth Health Information National Trends Survey (HINTS 5 cycle 1) conducted in the United States [52]. The National Cancer Institute in the United States managed the development of survey questions and survey administration [52]. Participants received the first mailing and reminder postcard along with a cover letter [52]. The cover letter states that the survey aims to understand individuals’ use of health information. In addition, the cover letter emphasizes the voluntary nature of participation. The survey was conducted through mail among individuals aged ≥18 years in the United States from January 25, 2017, to May 5, 2017 [52]. On the basis of the formula of the American Association for Public Opinion Research response rate 2, the overall response rate was 32.39%. In total, 3285 people completed the survey [52]. Those who had not met health professionals for their own care during the past year, except for emergency room visits, were excluded from the analyses. Ultimately, of the 3285 cases, 2823 (85.94%) were included in the study.

### Measures

#### EHR Use

On a 5-point scale (0=*0* to 4=≥*10 times*), respondents were asked, “How many times did you access your online medical record in the last 12 months?” (mean 0.63, SD 1.03) [53]. Multimedia Appendix 1 contains a copy of the survey, which includes all the questions used in the study.

#### Health Information Efficacy

Health information efficacy was measured on a 5-point scale (1=*completely confident* to 5=*not confident at all*) by asking participants to rate their confidence levels to obtain health-related advice or information when necessary [54-56]. Responses to the item were reversely coded so that higher scores indicate higher health information efficacy (mean 3.79, SD 0.92).

#### Patient-Centered Communication

Patient-centered communication was operationalized using 7 items adopted by several studies [3,57]. Respondents reported their experiences of patient-centered communication with health care professionals during the previous 12 months. Respondents were asked how often their health care providers did the following things on a 4-point scale (1=*always* to 4=*never*): (1) provided them with an opportunity to ask any question they had about health, (2) paid attention to their emotions and feelings, (3) engaged them in making decisions regarding their health care, (4) ensured that they understand things they should do to manage their health, (5) clarified things, (6) spent sufficient amount of time with them, and (7) assisted them in coping with uncertainty feelings regarding their health or medical care. Responses to the 7 items were reversely coded. Then, the recoded responses were averaged (Cronbach α=0.93; mean 3.39, SD 0.65).

#### Social Support

Social support items were adopted from the study by Emanuel et al [58]. Respondents were asked whether they had someone they could rely on for emotional support when they needed it, including talking over problems or assisting them in making difficult decisions. In addition, respondents were asked if they had any family members or friends to discuss their health. Each social support item was assessed on a 2-point scale (0=*no* and 1=*yes*). These 2 items showed a high correlation (*r*=0.52; *P*<.001). To investigate the moderating role of social support, we combined the responses from these 2 items into 1 dichotomous indicator. Participants who answered *yes* to both items were considered as those with high social support (2349/2823, 83.21%), while the others were classified as those with low social support (474/2823, 16.79%). Please note that classification into high and low social support groups is based on individuals’ self-perceptions regarding social support.

#### Control Variables

We included 3 types of control variables. First, age (mean 56.99, SD 15.92 years), male or female (1068/2763, 38.65% male), race or ethnicity (2037/2646, 76.98% White), education (1=<*8 years* to 7=*postgraduate*; mean 4.98, SD 1.59), income per year (1=<US $9999 to 9=≥US $200*,*000; mean 5.54, SD 2.25), employment status (1366/2744, 49.78% employed), marital status (1458/2727, 53.47% married), place of birth (2417/2758, 87.64% born in the United States), and having children (637/2574, 24.75% having children aged <18 years) were included as control variables. Second, we controlled for health-related variables: general health status (1=*poor* to 5=*excellent*; mean 3.35, SD 0.95), cancer history (471/2801, 16.82% ever been diagnosed as having cancer), regular health provider (2165/2787, 77.68% have health professionals that they see most often), and health insurance (2698/2787, 96.81% covered by health insurance). Third, we controlled for health information seeking from any source (2287/2768, 82.62% ever sought health information) because health information seeking from health care professionals or the media could increase health information efficacy or enhance the perceived quality of communication with physicians.

### Statistical Analyses

#### Mediation Analyses

We first examined whether health information efficacy mediates the relationship between EHR use and patient-centered communication. We conducted path analyses using Mplus (version 7.4) developed by Muthén and Muthén [59]. Our model included control variables significantly associated with endogenous variables at bivariate correlation analyses [60]: race or ethnicity, education, income per year, employment, place of birth, general health, regular health provider, health insurance, and health information seeking. The skewness and the kurtosis of the variables in the models indicate that all variables were normally distributed [61]. Unstandardized coefficients were reported following the recommendation of mediation analyses [62].

Following a methodology document made by the Health Information National Trends Survey (HINTS) [63], we used a sample weight and replicate weights. A sample weight corrects for oversampling and generalizes the result to the US population [63]. In addition, replicate weights allow researchers to recalculate SEs, which lowers the risk of committing a type 1 error [63].

#### Additional Analyses

One could argue that the directionality of the variables in our model may be reversed. That is, patient-centered communication might increase health information efficacy [57], or health information efficacy could promote EHR use. Through the model comparison analyses, we explored whether our hypothesized mediation model fits the data better than its competing models with different causal orders among variables [64,65]. Although a model comparison result does not support causal links among variables, we can compare the performance of each model.

**Figure 1:**
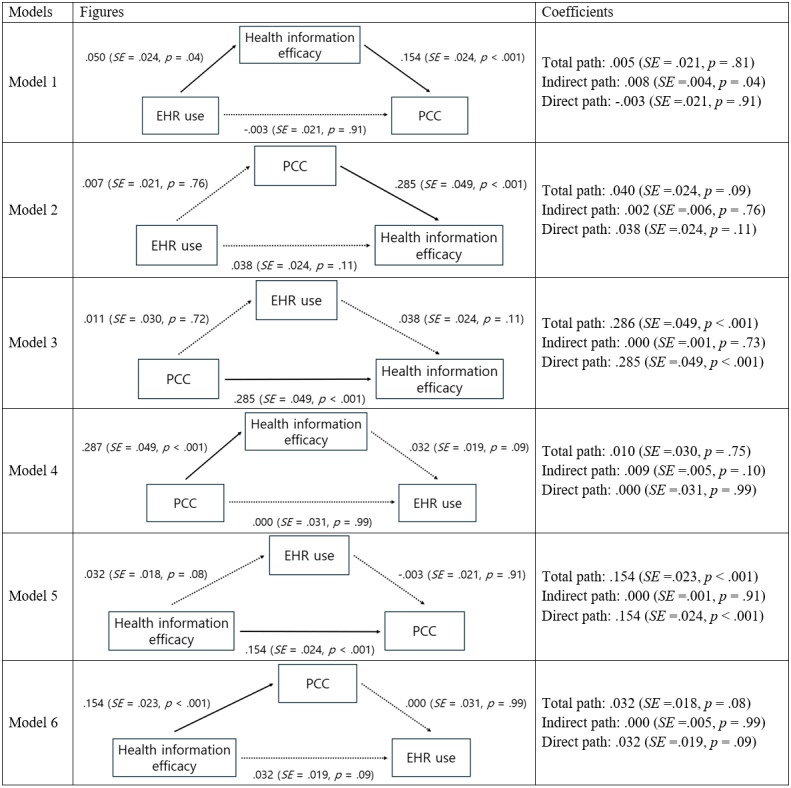
Comparison of mediation models. Unstandardized coefficients are shown with SEs and *P* values in parentheses. Paths with solid lines are statistically significant, while those with dotted lines are insignificant. Control variables are not presented in the figure. EHR: electronic health record; PCC: patient-centered communication.

As shown in Figure 1, we developed 5 alternative models. Model 2 assumes the indirect pathway from EHR use to health information efficacy through patient-centered communication. In model 3, EHR use mediates the relationship between patient-centered communication and health information efficacy. Model 4 posits that the relationship between patient-centered communication and EHR use is mediated by health information efficacy. In model 5, EHR use is a mediator for the path from health information efficacy to patient-centered communication. Model 6 assumes the indirect pathway from health information efficacy to EHR use via patient-centered communication. All models included control variables significantly associated with endogenous variables in the bivariate correlation analyses [60]. In models 1 and 2, where EHR use is an endogenous variable, EHR use was significantly related to race or ethnicity, education, income per year, employment, place of birth, general health, regular health provider, health insurance, and health information seeking at a bivariate level. Thus, these variables were included as control variables. In models 3 and 4, where patient-centered communication is an endogenous variable, patient-centered communication was significantly associated with race or ethnicity, income per year, place of birth, general health, regular health provider, and health insurance at a bivariate level. Therefore, these variables were controlled. In models 5 and 6, where health information efficacy is an endogenous variable, health information efficacy was significantly related to education, income per year, employment, place of birth, general health, and health information seeking at a bivariate level. These covariates were controlled in each model. In addition, we used a sample weight and replicate weights following the HINTS methodology document [63]. Figure 1 provides detailed information about additional models.

We used standard criteria for measuring fits to find the best-fitting model: (1) Akaike information criterion, (2) Bayesian information criterion, (3) root mean square error of approximation (RMSEA), and (4) standardized root mean squared residual (SRMR). Chi-square statistics were not used because Mplus does not provide it with replicate weights. We compared models based on each model’s average ranking across all fit statistics. The ranks of the 4 fit statistics of each model were averaged. A model with a high mean fit rank was considered better than its alternatives.

#### Multigroup Path Analyses

Next, we investigated whether and how social support moderates the linkage between EHR use and health information efficacy. A multigroup path analysis was conducted. We tested the difference of coefficients of the path from EHR use to health information efficacy between those who have high and low levels of social support. If the difference was significant, it was considered that the association of EHR use with health information efficacy differs by social support. We included the same set of control variables: race or ethnicity, education, income per year, employment, place of birth, general health, regular health provider, health insurance, and health information seeking. In addition, a sample weight and replicate weights were applied following the methodology document by the HINTS [63].

Finally, we conducted post hoc power analyses of variables (EHR use, health information efficacy, and patient-centered communication) for the comparability of the groups (high and low social support groups). First, we calculated effect sizes (Cohen *d*) for each variable between high and low social support groups. Then, we used G*Power (version 3.1) software, developed by Faul et al [66], to compute statistical power analyses.

### Ethical Considerations

This study analyzed data from the HINTS 5 cycle 1 survey. The Westat Institutional Review Board in the United States reviewed and approved the survey on March 28, 2016 (project number: 6048.14) [67]. Westat is a research firm that conducted the survey under contract with the United States Department of Health and Human Services. The survey received a “not human subjects research” determination from the National Institutes of Health Office of Human Subjects Research on April 25, 2016 (exempt number: 13204) [67]. Participants were invited to take the survey, receiving up to 3 mailings and 1 reminder postcard [63]. Each mail contains a cover letter, questionnaire, and return envelope. The cover letter informed participants that their participation was voluntary and their responses would not be linked to their names or any other information that could identify them or their households in accordance with the Privacy Act [63]. Survey data were stored with restricted access. Prepaid compensation of US $2 was provided to all potential participants to encourage participation [63].

## Results

### Descriptive Statistics

We presented descriptive statistics of all variables in our analyses in Table 1. Tables 2 and 3 also show bivariate correlations of the variables.

**Table 1:** Descriptive statistics.

		All respondents (N=2823)	Respondents with high social support^a^ (n=2349)	Respondents with low social support^a^ (n=474)
EHR^b^ use^c^, mean (SD)		0.63 (1.03)	0.65 (1.04)	0.52 (0.95)
HIE^d,e^, mean (SD)		3.79 (0.92)	3.85 (0.89)	3.47 (1.02)
PCC^f,g^, mean (SD)		3.39 (0.65)	3.45 (0.62)	3.12 (0.77)
Age (y), mean (SD)		56.99 (15.92)	56.93 (16.09)	57.10 (14.98)
Gender (male), n (%)		1068 (38.65)	884 (38.24)	171 (40.91)
Social support^a^ (high), n (%)		2349 (83.21)	—^h^	—
Race or ethnicity, n (%)				
	African American	486 (18.37)	396 (17.83)	83 (21.01)
	American Indian or Alaska Native	103 (3.89)	84 (3.78)	19 (4.81)
	Hispanic or Latinx	328 (12.78)	265 (12.31)	58 (14.99)
	White	2037 (76.98)	1725 (77.67)	289 (73.16)
Education^i^, mean (SD)		4.98 (1.59)	5.03 (1.59)	4.77 (1.56)
Income^j^ (per year), mean (SD)		5.54 (2.25)	5.68 (2.22)	4.85 (2.26)
Employment status, n (%)				
	Employed	1366 (49.78)	1154 (50.17)	197 (47.58)
	Retired	900 (32.8)	767 (33.35)	122 (29.47)
	Disabled	218 (7.94)	160 (6.96)	54 (13.04)
	Homemaker	148 (5.39)	126 (5.48)	21 (5.07)
	Unemployed	144 (4.15)	89 (3.87)	25 (6.04)
Marital status, n (%)				
	Married	1458 (53.47)	1286 (56.3)	158 (38.44)
	Divorced	435 (15.58)	327 (14.32)	90 (21.9)
	Single, never been married	416 (15.25)	324 (14.19)	86 (20.92)
	Widowed	283 (10.38)	233 (10.2)	47 (11.44)
Born in the United States, n (%)		2417 (87.64)	2030 (88.03)	359 (85.68)
Having children, n (%)		637 (24.75)	534 (24.68)	98 (25.39)
Health status^k^, mean (SD)		3.35 (0.95)	3.40 (0.93)	3.08 (1.04)
Cancer history, n (%)		471 (16.82)	403 (17.23)	68 (15.96)
With health provider, n (%)		2165 (77.68)	1841 (79.39)	294 (69.01)
Have health insurance, n (%)		2698 (96.81)	2257 (97.24)	400 (94.12)
HIS^l^ (seeking), n (%)		2287 (82.62)	1924 (83.47)	326 (77.99)

^a^Social support was operationalized by 2 items. Those who answered *yes* to both items were considered those with high social support. The others were regarded as those with low social support.

^b^EHR: electronic health record.

^c^EHR use was assessed on a 5-point scale: 0=*0* to 4*=≥10 times.*

^d^HIE: health information efficacy.

^e^HIE was measured on a 5-point scale: 1=*completely confident* to 5=*not confident at all*. Responses were reversely coded.

^f^PCC: patient-centered communication.

^g^Mean of 7 PCC items. Each item was measured on a 4-point scale: 1=*always* to 4=*never*. Responses were reversely coded.

^h^Not available.

^i^Education was assessed on a 7-point scale: 1=<*8 years* to 7*=postgraduate*. Responses were averaged.

^j^Income per year was measured on a 9-point scale: 1=*<US $9999* to 9=≥*US $200,000*. Responses were averaged.

^k^General health status was measured on a 5-point scale: 1=*poor* to 5=*excellent*.

^l^HIS: health information seeking.

**Table 2:** Bivariate correlations of variables.

			EHR^a^		HIE^b^		PCC^c^		Social support		Age		Male or female		Race or ethnicity		Education
HIE																	
	*r*	0.078		—^d^		—		—		—		—		—		—	
	*P* value	<.001		—		—		—		—		—		—		—	
PCC																	
	*r*	0.066		0.242		—		—		—		—		—		—	
	*P* value	.01		<.001		—		—		—		—		—		—	
Social support																	
	*r*	0.030		0.154		0.117		—		—		—		—		—	
	*P* value	.23		<.001		<.001		—		—		—		—		—	
Age																	
	*r*	−0.004		−0.026		0.045		0.098		—		—		—		—	
	*P* value	.90		.50		.21		.006		—		—		—		—	
Male or female																	
	*r*	−0.050		−0.008		0.002		−0.019		0.004		—		—		—	
	*P* value	.10		.79		.96		.58		.89		—		—		—	
Race or ethnicity																	
	*r*	0.066		0.021		0.119		0.069		0.115		0.012		—		—	
	*P* value	.006		.49		<.001		.04		<.001		.59		—		—	
Education																	
	*r*	0.199		0.066		0.063		0.126		−0.058		−0.038		0.166		—	
	*P* value	<.001		.03		.06		<.001		.13		.03		<.001		—	
Income per year																	
	*r*	0.184		0.131		0.093		0.133		−0.010		0.088		0.117		0.361	
	*P* value	<.001		<.001		.01		<.001		.79		.01		<.001		<.001	
Employment status																	
	*r*	0.094		0.089		0.015		0.046		−0.252		0.091		0.029		0.260	
	*P* value	<.001		.006		.66		.22		.001		.008		.30		<.001	
Marital status																	
	*r*	0.130		0.037		0.035		0.093		0.203		0.056		0.140		0.118	
	*P* value	<.001		.25		.20		.003		<.001		.001		<.001		<.001	
Born in the United States																	
	*r*	0.010		0.069		0.136		0.038		0.137		−0.011		0.304		0.007	
	*P* value	.70		.01		<.001		.33		<.001		.76		<.001		.84	
Having children aged <18 years																	
	*r*	0.035		0.011		−0.018		−0.078		−0.219		−0.069		−0.030		0.015	
	*P* value	.18		.73		.53		.05		<.001		.02		.22		.67	
General health																	
	*r*	0.043		0.153		0.147		0.213		−0.137		−0.003		0.082		0.267	
	*P* value	.12		<.001		<.001		<.001		.001		.94		.003		<.001	
Cancer history																	
	*r*	0.030		−0.036		−0.004		0.006		0.241		−0.046		0.028		−0.024	
	*P* value	.19		.09		.82		.79		<.001		.03		.16		.13	
Regular health provider																	
	*r*	0.192		0.034		0.194		0.063		0.217		−0.040		0.157		0.044	
	*P* value	<.001		.25		<.001		.05		<.001		.30		<.001		.13	
Health insurance																	
	*r*	0.101		0.030		0.192		0.024		0.096		0.007		0.070		0.140	
	*P* value	<.001		.56		<.001		.50		.004		.88		.09		<.001	
HIS^e^																	
	*r*	0.166		0.138		0.035		0.077		−0.010		−0.029		0.109		0.261	
	*P* value	<.001		.002		.20		.04		.81		.38		<.001		<.001	

^a^EHR: electronic health record.

^b^HIE: health information efficacy.

^c^PCC: patient-centered communication.

^d^Not available.

^e^HIS: health information seeking.

**Table 3:** Additional bivariate correlations of variables.

			Income per year		Employment status		Marital status		Born in the United States		Having children aged <18 years		General health		Cancer history		Regular health provider		Health insurance
Income per year																			
	*r*	—^a^		—		—		—		—		—		—		—		—	
	*P* value	—		—		—		—		—		—		—		—		—	
Employment status																			
	*r*	0.393		—		—		—		—		—		—		—		—	
	*P* value	<.001		—		—		—		—		—		—		—		—	
Marital status																			
	*r*	0.344		0.160		—		—		—		—		—		—		—	
	*P* value	<.001		<.001		—		—		—		—		—		—		—	
Born in the United States																			
	*r*	0.024		−0.079		−0.033		—		—		—		—		—		—	
	*P* value	.41		.007		.29		—		—		—		—		—		—	
Having children aged <18 years																			
	*r*	0.149		0.195		0.210		−0.086		—		—		—		—		—	
	*P* value	<.001		<.001		<.001		.02		—		—		—		—		—	
General health																			
	*r*	0.225		0.200		0.089		−0.003		0.034		—		—		—		—	
	*P* value	<.001		<.001		.007		.95		.23		—		—		—		—	
Cancer history																			
	*r*	−0.004		−0.128		0.055		0.030		−0.077		−0.074		—		—		—	
	*P* value	.85		<.001		.27		.08		<.001		.001		—		—		—	
Regular health provider																			
	*r*	0.060		−0.084		0.131		0.157		−0.075		−0.047		0.095		—		—	
	*P* value	.14		.03		<.001		<.001		.04		.17		<.001		—		—	
Health insurance																			
	*r*	0.075		0.064		0.082		−0.003		−0.055		0.012		0.045		0.217		—	
	*P* value	.07		.03		.003		.92		.25		.69		.002		<.001		—	
HIS^b^																			
	*r*	0.164		0.124		0.099		0.022		−0.017		0.070		0.057		0.043		0.074	
	*P* value	<.001		<.001		.007		.44		.60		.04		.001		.20		.12	

^a^Not available.

^b^HIS: health information seeking.

### Mediation Analyses

We conducted path analyses to investigate whether health information efficacy mediates the relationship between EHR use and patient-centered communication. Our mediation model fit the data well (RMSEA=0.015, 90% CI 0.000-0.031; SRMR=0.006). EHR use was positively associated with health information efficacy (unstandardized coefficient=0.050, SE 0.024; *P*=.04). In turn, health information efficacy was positively linked to patient-centered communication (unstandardized coefficient=0.154, SE 0.024; *P*<.001). The indirect pathway hypothesized in our study was statistically significant: health information efficacy mediated the association of EHR use with patient-centered communication (unstandardized coefficient=0.008, SE 0.004; *P*=.04).

### Additional Analyses

Next, we conducted model comparison analyses. We compared our hypothesized mediation model (model 1) with its competing 5 models (models 2-6). Table 4 shows 4 fit statistics (Akaike information criterion, Bayesian information criterion, RMSEA, and SRMR) and the rank of each fit statistic. The average rank of our hypothesized model (model 1) was 1.5, which is the best among the 6 plausible models.

**Table 4:** Comparison of the fit measures for the 6 models, with the rank order of fit indicated.

Models	AIC^a^			BIC^b^			RMSEA^c^			SRMR^d^			Rank, mean (SD)
	Values	Rank	Values		Rank	Values		Rank	Values		Rank		
Model 1^e^	68,812.127	1	69,311.553		1	0.015		1	0.006		3	1.5 (0.87)	
Model 2^f^	68,813.358	2	69,312.785		2	0.017		6	0.006		3	3.25 (1.64)	
Model 3^g^	72,461.655	6	73,044.319		6	0.016		5	0.006		3	5 (1.22)	
Model 4^h^	72,461.418	5	73,044.082		5	0.015		1	0.006		3	3.5 (1.66)	
Model 5^i^	72,461.026	3	73,043.690		3	0.015		1	0.005		1	2 (1.00)	
Model 6^j^	72,461.054	4	73,043.718		4	0.015		1	0.005		1	2.5 (1.50)	

^a^AIC: Akaike information criterion.

^b^BIC: Bayesian information criterion.

^c^RMSEA: root mean square error of approximation.

^d^SRMR: standardized root mean squared residual.

^e^Our hypothesized model.

^f^Electronic health record use→patient-centered communication→health information efficacy.

^g^Patient-centered communication→electronic health record use→health information efficacy.

^h^Patient-centered communication→health information efficacy→electronic health record use.

^i^Health information efficacy→electronic health record use→patient-centered communication.

^j^Health information efficacy→patient-centered communication→electronic health record use.

### Multigroup Path Analyses

Furthermore, we conducted a multigroup path analysis to explore the moderating role of social support in the relationship between EHR use and health information efficacy. The model with a total sample showed a good fit (RMSEA=0.030, 90% CI 0.016-0.045; SRMR=0.010). In addition, the model fit the data well in each group: high social support group (RMSEA=0.030, 90% CI 0.015-0.046; SRMR=0.009) and low social support group (RMSEA=0.033, 90% CI 0.000-0.074; SRMR=0.013). The path coefficients of the association of EHR use with health information efficacy were significantly different between high and low social support groups (unstandardized coefficient=0.118, SE 0.058; *P*=.04).

Among patients with high social support, EHR use was not significantly related to health information efficacy (unstandardized coefficient=0.038, SE 0.026; *P*=.15). By contrast, health information efficacy was positively associated with patient-centered communication (unstandardized coefficient=0.151, SE 0.030; *P*<.001). The indirect pathway from EHR use to patient-centered communication through health information efficacy was not significant among those who have high levels of social support (unstandardized coefficient=0.006, SE 0.004; *P*=.11).

**Figure 2:**
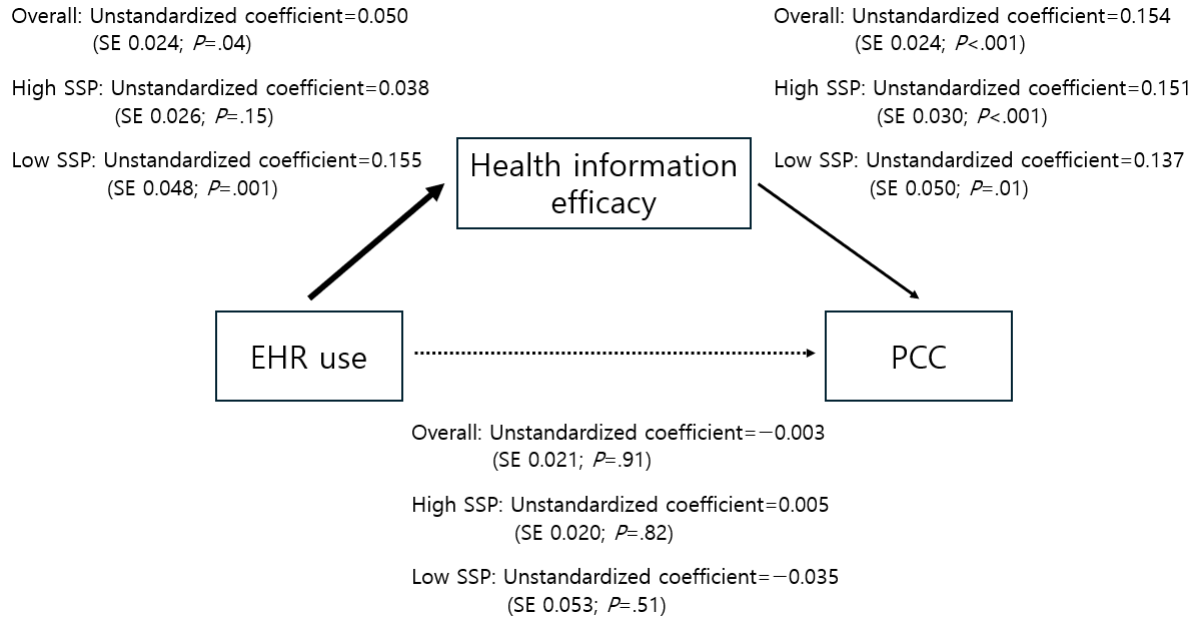
A multigroup path analysis predicting high and low social support (SSP) groups’ patient-centered communication (PCC). Unstandardized coefficients are shown with SEs and *P* values in parentheses. Coefficients for all participants are first reported, followed by coefficients for those with high SSP and low SSP. Paths with solid lines are statistically significant, while those with dotted lines are insignificant. In addition, bold lines indicate significant differences between those with high and low SSP. Control variables are not presented in the figure. The model fits the data well (root mean square error of approximation=0.030, 90% CI 0.016-0.045; standardized root mean squared residual=0.010). EHR: electronic health record.

However, among patients with low social support, EHR use was positively related to health information efficacy (unstandardized coefficient=0.155, SE 0.048; *P*=.001), which in turn was positively associated with patient-centered communication (unstandardized coefficient=0.137, SE 0.050; *P*=.01). Health information efficacy was a significant mediator for the path from EHR use to patient-centered communication among those with low levels of social support (unstandardized coefficient=0.021, SE 0.010; *P*=.03). The relationship among variables with unstandardized coefficients is presented in Figure 2.

Finally, we conducted post hoc power analyses of variables to explore the comparability of high and low social support groups. Results show an effect size (Cohen *d*) of 0.126 for EHR use, 0.408 for health information efficacy, and 0.512 for patient-centered communication. In addition, findings from post hoc power analyses with a 2-tailed *t* test and an α level of .05 show that the power is 66.71% for EHR use, 100% for health information efficacy, and 100% for patient-centered communication.

## Discussion

### Principal Findings

The existing literature pays little attention to the underlying mechanism of how patients’ EHR use is beneficial to patient-centered communication. We found that health information efficacy, to some extent, accounts for the linkage between EHR use and patient-centered communication. The results indicate that EHR use seems to increase patients’ beliefs that they can obtain necessary health information. In turn, patients with health information efficacy have patient-centered communication with health care professionals, probably because these patients receive the information they desire from physicians while asking questions and become informed of their health, and they thus easily participate in medical decision-making. In addition, EHR use was positively associated with health information efficacy, and we detected such a relationship only among patients who lack social support. Patients who feel they do not receive support from others may meet their needs through the use of EHRs.

Prior research in this field, most of which are qualitative studies, reported mixed findings regarding the relationship between EHR use and patient-centered communication. While EHRs enable patients to communicate with physicians effectively [14,17], there is a possibility that EHR use can interfere with patient-centered communication for certain individuals. One of the challenges associated with EHR systems is maintaining the privacy of patient information [19,68,69]. Concerns about the confidentiality of health records stored in EHRs may discourage patients from sharing their health status with physicians [2,68]. Such concerns might prevent patients from communicating with health professionals. Furthermore, some patients still struggle to understand medical terminologies or interpret test results in EHRs [70]. In addition, health care professionals often experience stress due to the high volume of messages they receive from patients through EHRs [71], which might compromise the quality of communication between physicians and patients. Thus, future studies should adopt a more nuanced approach by considering who uses EHRs in what contexts and what characteristics of EHRs could act as barriers to patient-centered communication. It would be worthwhile to explore further whether the impact of EHR use on patient-centered communication differs according to patients’ levels of security concerns or physicians’ proficiency in managing eHealth technologies.

### Limitations

There are some limitations in this study. First, we could not establish a causal relationship among EHR use, health information efficacy, and patient-centered communication because the survey was cross-sectional. Second, we measured EHR use and health information efficacy using only 1 item. A single-item measure could result in measurement errors, which may reduce the chances of obtaining statistically significant associations. Third, unmeasured factors, such as patients’ personality traits or medical mistrust, could affect patients’ communication experiences with health professionals [1]. Fourth, this study relies on self-reported perceptions of patient-centered communication. There is the possibility of recall bias in the self-reported measures [72-74]. While self-reported measures of patient-centered communication assess patients’ overall evaluations of their interactions with health professionals [3,72,75-77], these measures do not allow researchers to investigate what specific types of patients or health professionals’ behaviors influence such assessments of patient-centered communication [75]. To address these limitations, scholars have suggested the use of observational measures, such as training coders to review video recordings of patients consulting with health professionals [72,75,77]. Future research may benefit from using both self-reported and observational measures of patient-centered communication within the same study. Fifth, categorization into high and low social support groups relies on individuals’ self-perceptions of social support. Because perceived social support measures may be influenced by perceptual or judgmental differences among individuals [78], future studies could use other measures of social support, such as received social support that captures specific behaviors or actions provided to individuals [78]. Sixth, previous studies referred to questions asking about patients’ web-based medical record use in the HINTS as EHR use [53,79-82]. However, the HINTS items did not specifically ask what type of web-based health record patients accessed, specifically whether patients used EHRs, EMRs, or PHRs, which could limit the generalizability of the results. Finally, the findings of this study are relevant to the United States. Future studies could explore how the relationship between web-based medical record use and patient-centered communication varies across countries.

### Implications

Our study has several theoretical implications. First, we empirically investigated the mechanism through which EHR use by patients could improve the quality of communication between health professionals and patients. Drawing from the self-efficacy theory, we explicate how health information efficacy links the relationship between EHR use and patient-centered communication. Moreover, this study suggests that EHRs can compensate for a lack of social support by enhancing beliefs in one’s ability to acquire health information. Existing studies show that social capital, resources embedded within one’s social network, could increase the use of diverse health information sources [83] and frequent health information seeking [30,31]. This means that the resources that are obtained through social relationships may have differential influences on health behaviors. However, our study suggests that EHR use may help bridge these gaps. Patients who lack social support could derive benefits from EHRs, which might improve the quality of patient-centered communication.

This study has practical implications as well. First, it is important for patients to develop efficacy beliefs about health information acquisition. According to the literature on self-efficacy, individuals with a high sense of efficacy set high goals and commit to them [34]. Patients with health information efficacy might aim to acquire health information from health providers and prepare for meetings with them. Thus, enhancing patients’ health information efficacy should be a crucial element in patient education and public health interventions. By boosting efficacy beliefs, health educators and public health professionals may successfully enhance communication between patients and physicians.

Furthermore, EHR use is beneficial for patients, especially those with low social support, in building health information efficacy. However, patients’ access to health records on the web remains low, although most hospitals in the United States have already adopted EHRs [84,85]. Similarly, of those who have met health professionals over the past year, 65.3% (1794/2748) of respondents have never had access to EHRs, according to the HINTS data we analyzed. Given that physicians’ recommendation for EHR use is the main factor promoting patients’ EHR use [53], health care providers should encourage patients to use EHRs.

### Conclusions

To understand the pathway from patients’ use of EHRs to patient-centered communication, we investigated the mediating role of health information efficacy. We used the data from the HINTS 5 cycle 1, which was a survey with a nationally representative sample, and the statistical weighting makes our results generalizable to the US population aged ≥18 years. Our findings indicate that EHR use by patients appears to promote health information efficacy, which may in turn facilitate patient-centered communication. This indirect relationship was not detected among patients with high social support. However, among patients with low social support, EHR use seems to enhance health information efficacy, which may subsequently promote patient-centered communication. These findings are more relevant to the United States than other contexts. In addition, using a cross-sectional survey prevents us from making strong causal claims about any relationship reported in our study.

## Appendix

Multimedia Appendix 1 Copy of the survey including all the questions used in the study.

## Abbreviations

EHR: electronic health record
EMR: electronic medical record
HINTS: Health Information National Trends Survey
PHR: personal health record
RMSEA: root mean square error of approximation
SRMR: standardized root mean squared residual

## Data Availability

The datasets generated during and analyzed during this study are available in the Health Information National Trends Survey repository [52].
