# Supplementary material for: Patients’ Use of Electronic Health Records Facilitates Patient-Centered Communication: Findings From the 2017 Health Information National Trends Survey
Source: J Med Internet Res. 2024 Nov 25;26:e50476. doi: 10.2196/50476 (PMC11629042; doi:10.2196/50476)
Supplement: Multimedia Appendix 1 [file jmir_v26i1e50476_app1.docx]

Multimedia Appendix 1 for

"Patients’ Use of Electronic Health Records Facilitates Patient-Centered Communication: Findings From the 2017 Health Information National Trends Survey"

Appendix A. Questionnaire items

* Survey questions in the Cycle 1 of the fifth Health Information National Trends Survey (HINTS)

1. Electronic health record (EHR) use

D4. How many times did you access your online medical record in the last 12 months?

1. 0
2. 1 to 2 times
3. 3 to 5 times
4. 6 to 9 times
5. 10 or more times

2. Health information efficacy

A5. Overall, how confident are you that you could get advice or information about health or medical topics if you needed it?

1. Completely confident
2. Very confident
3. Somewhat confident
4. A little confident
5. Not confident at all

3. Patient-centered communication

C4. The following questions are about your communication with all doctors, nurses, or other health professionals you saw during the past 12 months. How often did they do each of the following:

1. Give you the chance to ask all the health-related questions you had.
2. Give the attention you needed to your feelings and emotions.
3. Involve you in decisions about your health care as much as you wanted.
4. Make sure you understood the things you needed to do to take care of your health.
5. Explain things in a way you could understand.
6. Spend enough time with you.
7. Help you deal with feelings of uncertainty about your health or health care.

Response options:

1. Always
2. Usually
3. Sometimes
4. Never

4. Social support

G7. Is there anyone you can count on to provide you with emotional support when you need it - such as talking over problems or helping you make difficult decisions?

1. Yes
2. No

G8. Do you have friends or family members that you talk to about your health?

1. Yes
2. No

*** No. 5 – 18 were used as control variables.**

5. Age

O1. What is your age? *[open-ended]*

6. Gender

K1. Are you male or female?

1. Male
2. Female

7. Race/ethnicity

O10. Are you of Hispanic, Latino/a, or Spanish origin? One or more categories may be selected. Mark all that apply.

1. No, not of Hispanic, Latino/a, or Spanish origin
2. Yes, Mexican, Mexican American, Chicano/a
3. Yes, Puerto Rican
4. Yes, Cuban
5. Yes, another Hispanic, Latino/a, or Spanish origin

O11. What is your race? One or more categories may be selected. Mark all that apply.

1. White
2. Black or African American
3. American Indian or Alaska Native
4. Asian Indian
5. Chinese
6. Filipino
7. Japanese
8. Korean
9. Vietnamese
10. Other Asian
11. Native Hawaiian
12. Guamanian or Chamorro
13. Samoan
14. Other Pacific Islander

8. Education

O6. What is the highest grade or level of schooling you completed? Education

1. Less than 8 years
2. 8 through 11 years
3. 12 years or completed high school
4. Post high school training other than college (vocational or technical)
5. Some college
6. College graduate
7. Postgraduate

9. Income per year

O19. Thinking about members of your family living in this household, what is your combined annual income, meaning the total pre-tax income from all sources earned in the past year?

1. $0 to $9,999
2. $10,000 to $14,999
3. $15,000 to $19,999
4. $20,000 to $34,999
5. $35,000 to $49,999
6. $50,000 to $74,999
7. $75,000 to $99,999
8. $100,000 to $199,999
9. $200,000 or more

10. Employment status

O2. What is your current occupational status? **Mark only one**.

1. Employed
2. Unemployed
3. Homemaker
4. Student
5. Retired
6. Disabled
7. Other-Specify *[open-ended]*

11. Marital status

O5. What is your marital status? **Mark only one**.

1. Married
2. Living as married
3. Divorced
4. Widowed
5. Separated
6. Single, never been married

12. Place of birth

O7. Were you born in the United States?

1. Yes
2. No

13. Having children

O15. How many children under the age of 18 live in your household? *[open-ended]*

14. General health status

G1. In general, would you say your health is ...

1. Excellent
2. Very good
3. Good
4. Fair
5. Poor

15. Cancer history

M1. Have you ever been diagnosed as having cancer?

1. Yes
2. No

16. Regular health provider

C1. Not including psychiatrists and other mental health professionals, is there a particular doctor, nurse, or other health professional that you see most often?

1. Yes
2. No

17. Health insurance

C7. Are you currently covered by any of the following types of health insurance or health coverage plans?

a. Insurance through a current or former employer or union.

b. Insurance purchased directly from an insurance company.

c. Medicare, for people 65 and older, or people with certain disabilities.

d. Medicaid, Medical Assistance, or any kind of government-assistance plan for those with low incomes or a disability.

e. TRICARE or other military health care.

f. VA (including those who have ever used or enrolled for VA health care).

g. Indian Health Service.

h. Any other type of health insurance or health coverage plan (specify). *[open-ended]*

Response options:

1. Yes
2. No

18. Health information seeking

A1. Have you ever looked for information about health or medical topics from any source?

1. Yes
2. No
